# Airborne particulates and brain health: The role of PM_2.5_ in blood–brain-barrier dysfunction

**DOI:** 10.1177/0271678X261418925

**Published:** 2026-02-08

**Authors:** Fátima Gimeno-Ferrer, Lisa Teresa Porschen, Frank Matthes, Katrin Gohlsch, Anja Meissner

**Affiliations:** 1 Division of Physiology & Vascular Biology, Faculty of Medicine, Institute of Theoretical Medicine, University of Augsburg, Augsburg, Germany; 2 Department of Experimental Medical Science, Faculty of Medicine, Lund University, Lund, Sweden; 3 Faculty of Medicine, Wallenberg Center for Molecular Medicine, Lund University, Lund, Sweden; 4 Model-Based Environmental Exposure Science, Faculty of Medicine, University of Augsburg, Augsburg, Germany

**Keywords:** Particulate matter, blood–brain barrier, neuroinflammation, oxidative stress

## Abstract

Ambient particulate matter (PM), especially fine and ultrafine particles, has emerged as a significant environmental risk factor for neurological disorders, largely through its impact on the blood–brain barrier (BBB) and the neurovascular unit. This review summarizes current evidence on how PM affects BBB integrity, emphasizing the coordinated and cell-specific responses that drive neurovascular dysfunction. Upon systemic or neural translocation, PM induces oxidative stress and inflammation in endothelial cells, disrupting tight junctions (TJs), enhancing permeability, and upregulating adhesion molecules (e.g. ICAM-1 and VCAM-1), which facilitate immune cell infiltration. Pericytes contribute to these processes in a stage-dependent manner, promoting BBB leakage through detachment and inflammation in acute settings while participating in later reparative processes such as angiogenesis and neurogenesis. Astrocytes respond to PM exposure by adopting a reactive phenotype, releasing pro-inflammatory cytokines and reactive oxygen species that exacerbate barrier disruption and impair neurovascular coupling. Microglia act as central mediators of neuroinflammation, releasing cytokines that weaken TJs and perpetuate endothelial dysfunction. These mechanisms are further modulated by particle properties and host-related factors including age, metabolic status, and pre-existing comorbidities. The resulting cascade of BBB impairment and neuroinflammation underscores the multifaceted nature of PM-induced neurotoxicity and identifies potential cellular targets for intervention.

## Introduction

Air pollution represents a significant global health concern, with particulate matter (PM) recognized as a major contributing factor.^1,2^ Particulate matter comprises a complex mixture of solid and liquid particles suspended in the air, including dust, soot, smoke, and organic and inorganic compounds.^3,4^ These particles are categorized based on their aerodynamic diameter into coarse (PM_10_), fine (PM_2.5_), and ultrafine (PM_0.1_) particles. Numerous epidemiological studies have established strong associations between PM exposure and adverse health outcomes, especially in the cardiovascular and respiratory systems.^5–7^ However, emerging evidence suggests that finer fractions of PM, especially fine (PM_2.5_) and ultrafine (PM_0.1_) particles, pose significant risks to the central nervous system (CNS),^8^ potentially contributing to cerebrovascular diseases, neurodegenerative diseases and cognitive dysfunction^9,10^ due to their ability to interact with and penetrate biological barriers more effectively. Finer particles can accumulate and propagate longer distances and, therefore, they remain longer in the atmosphere and become persistent compared to larger particles.^8,11–14^

A critical interface between the peripheral circulation and the CNS is the blood–brain barrier (BBB), which dynamically regulates the passage of molecules, ions, and cells between the bloodstream and the brain.^15^ It plays a vital role in regulating oxygen and nutrient supply, maintaining CNS homeostasis and protecting neural tissue from toxins and pathogens. Disruption of BBB integrity can lead to increased permeability, allowing harmful substances to enter the brain and trigger neuroinflammation, oxidative stress, and neuronal damage.^16^ Recent studies have indicated that exposure to fine and ultrafine PM may compromise BBB function through direct and indirect mechanisms, involving inflammatory pathways, oxidative damage, and alterations in BBB cell function.^17–19^ It is likely that the adverse effects of PM exposure may manifest at the BBB over an extended period before clinical symptoms are observed.

This review summarizes the knowledge on the impact of particularly PM_2.5_ on the BBB, highlighting the currently known underlying mechanisms of BBB disruption, neuroinflammation and the potential neurological consequences. Gaining insight into the interactions between PM-derived trigger substances and the various BBB constituents is vital for understanding how environmental pollutants impact health status and contribute to diseases with neurological trajectories, enabling the development of therapeutic strategies to mitigate their detrimental effects on brain health.

## Composition and function of the blood–brain barrier in health and disease

The BBB is a specialized, semi-permeable border that protects the CNS by regulating the entry of substances from the bloodstream into the brain. Structurally, it is formed by tightly connected brain microvascular endothelial cells, supported by a basement membrane, pericytes, and astrocytic end-feet. Tight junction (TJ) proteins such as claudins, occludins, and junctional adhesion molecules reinforce the BBB’s low paracellular permeability. Endothelial cells manage the selective transport of nutrients and waste, while pericytes maintain vascular stability and BBB integrity. Astrocytes secrete trophic and signaling factors that modulate endothelial function and BBB permeability. Microglia, as the brain’s innate immune sentinels, contribute to homeostasis through immune surveillance and clearance of debris.^20,21^ Under healthy conditions, this cellular architecture maintains a stable neural environment, ensuring tightly regulated exchange of ions, metabolites, and signaling molecules. However, in pathological states, each component of the BBB can be compromised.^22^ Inflammation and oxidative stress are key drivers of BBB disruption across diverse neurological conditions, impairing endothelial cell function and promoting barrier leakage, immune cell infiltration, and neuroinflammatory cascades that contribute to neuronal injury and cognitive decline.^23–25^ Progressive vascular damage links BBB disruption to the progression of dementia,^26^ classical neurodegenerative diseases, including Alzheimer’s disease (AD),^27^ Parkinson’s disease (PD),^28,29^ and neurodegenerative and neuroinflammatory trajectories related to cardiometabolic disease,^30,31^ underscoring the importance of vascular health in maintaining cognitive function.^32^

Endothelial cells form the anatomical and functional core of the BBB, lining cerebral capillaries with a continuous monolayer sealed by complex TJs. These junctions restrict paracellular diffusion and maintain the highly selective permeability of the barrier. In addition to their structural role, endothelial cells mediate the regulated transport of nutrients, ions, and waste products between the blood and the CNS, contributing to the homeostatic microenvironment required for neuronal function. In health, endothelial cells actively contribute to neurovascular integrity by sensing hemodynamic and metabolic signals, maintaining low transcytotic activity, and expressing selective transport systems. They also engage in crosstalk with pericytes, astrocytes, and microglia to coordinate barrier stability and CNS immune surveillance. In disease, endothelial dysfunction is an early and central event in BBB breakdown. It is characterized by disrupted TJ protein expression, increased oxidative stress, and heightened inflammatory responsiveness. Inflammatory stimuli, including circulating cytokines and pathogen-associated molecular patterns (PAMPs), induce endothelial activation, which is marked by the upregulation of adhesion molecules such as intercellular adhesion molecule-1 (ICAM-1) and vascular cell adhesion molecule-1 (VCAM-1). These molecules facilitate leukocyte adhesion and extravasation.^33^ This inflammatory priming increases BBB permeability and promotes the infiltration of peripheral immune cells into the CNS. In AD, endothelial dysfunction is associated with diminished TJ integrity and enhanced vascular deposition of amyloid-beta, contributing to cerebral amyloid angiopathy and neurodegeneration.^34,35^ The current knowledge about endothelial dysfunction in PD is limited, but a recent study found an enlargement of perivascular space, an increased presence of string vessels, which may impact on the blood flow, and a loss of endothelial cell, postulating an endothelial degeneration in PD with yet unclear mechanism.^36^ Similarly, in the context of cardiometabolic disorders like hypertension and diabetes, chronic endothelial activation results in impaired nitric oxide (NO) signaling, elevated reactive oxygen species (ROS), and vascular rarefaction.^37–39^ In ischemic stroke, endothelial-derived matrix metalloproteinases (MMPs), particularly MMP-9, are released in response to ischemia and degrade TJ and basement membrane components, further destabilizing the barrier.^40,41^ In diabetic mice, this response is exacerbated, leading to enhanced degradation of TJ protein and collagen IV, resulting in increased BBB permeability and greater neutrophil infiltration in the infarcted area.^42^ These processes collectively contribute to cerebral edema, neuroinflammation, and secondary neuronal injury.

Pericytes play pivotal and multifaceted roles in maintaining BBB integrity, cerebrovascular stability, and neurovascular unit (NVU) function. Situated within the basement membrane and closely associated with endothelial cells, they contribute to vascular stability, control capillary diameter, and modulate endothelial proliferation and permeability.^43^ In pathological states, pericyte dysfunction or loss is increasingly recognized as a key contributor to BBB disruption and disease progression.^32^ During the acute phase of ischemic stroke, pericytes detach from the vessel wall and adopt to a contractile and inflammatory phenotype, releasing cytokines and MMPs that degrade TJs and exacerbate BBB permeability.^44,45^ These changes contribute to reduced microvascular perfusion and increased neuroinflammation. In the recovery phase, however, pericytes exert neuroprotective effects by supporting endothelial repair, promoting angiogenesis through vascular endothelial growth factor (VEGF) signaling, and contributing to neurogenesis and tissue remodeling.^44,45^ Similarly, in neurodegenerative diseases such as AD, pericyte loss has been associated with reduced cerebral blood flow, impaired clearance of amyloid-beta, and increased vascular permeability, which collectively contribute to cognitive decline and neurovascular dysfunction.^46^ Recent research also point towards a lower density of pericytes on capillaries and their absence on string vessels in PD.^36^ Pericyte dysfunction is also observed in metabolic disorders such as diabetes and obesity, where chronic hyperglycemia, oxidative stress, and inflammation drive pericyte loss.^47^ This destabilizes the microvasculature, compromises endothelial barrier function, and promotes BBB breakdown. In diabetic encephalopathy and other forms of small vessel disease, pericyte degeneration further contributes to white matter damage and impaired neurovascular coupling.^48^ While many studies support a central role for pericytes in regulating BBB integrity, recent work has highlighted that the relationship between pericyte loss and barrier permeability may not be universal. Focal pericyte ablation in vivo was reported to lead to capillary dilation without measurable increases in BBB permeability.^49^ This finding suggests that the influence of pericytes on barrier function may be context-dependent, influenced by the extent and regional distribution of pericyte loss, compensatory endothelial mechanisms, or differential pericyte heterogeneity across vascular segments. Taken together, these findings underscore both the essential and dynamic roles of pericytes in BBB regulation and the need for further studies to clarify under which conditions pericyte loss translates into functional barrier disruption.

Additionally, astrocytes critically regulate BBB function and CNS homeostasis.^50^ Their end-feet enwrap the cerebral vasculature and secrete trophic and regulatory factors that support endothelial TJ integrity, modulate ion and neurotransmitter balance, and facilitate metabolic exchange between blood and brain. In health, astrocytes contribute to neurovascular coupling, maintain extracellular glutamate levels through excitatory amino acid transporters, and support the glymphatic clearance system via aquaporin-4 (AQP4) water channels.^29^ In response to injury, metabolic stress, or inflammation, astrocytes undergo a phenotypic transformation into a reactive state, including the neurotoxic A1 phenotype. Reactive astrocytes release pro-inflammatory cytokines (e.g. interleukin 1β (IL-1β) and tumor necrosis factor α (TNF-α)) and ROS, which can impair endothelial function and disrupt TJs, thereby compromising BBB integrity.^51^ In neurodegenerative diseases, alterations in astrocytic AQP4 localization and function have been linked to impaired glymphatic clearance in PD and AD, and enhanced amyloid-beta accumulation in AD.^28,29,52^ Moreover, reactive astrocytes contribute to oxidative stress, reduced glutamate uptake, and neurovascular uncoupling, exacerbating neuronal dysfunction and cognitive decline.^53,54^ In cardiometabolic disorders, chronic astrocyte activation has been associated with insulin resistance in the brain, elevated inflammatory signaling, and impaired cerebral blood flow regulation.^55–57^ In ischemic stroke, astrocytes exhibit context-dependent roles: acutely, they may worsen injury via the release of cytotoxic mediators, while during the recovery phase, they contribute to repair by producing neurotrophic factors, anti-inflammatory cytokines, and supporting synaptic remodeling.^58,59^ In AD^60^ and PD,^61^ astrocytes similarly become reactive in response to ROS, neuroinflammation, synaptic dysfunction, and neuronal apoptosis. Their response is biphasic, initially beneficial through compensatory mechanisms that help preserve neuronal function, but potentially detrimental when exacerbated, ultimately contributing to the progression of neurodegeneration. These dual roles highlight the importance of astrocytes as both protective and pathogenic contributors to BBB function and CNS pathology.

Microglia as the resident immune cells of the CNS serve as sentinels of the neural environment.^62^ In the healthy brain, they continuously survey the parenchyma, phagocytose cellular debris, and contribute to synaptic pruning and immune regulation. They also support BBB maintenance by modulating endothelial barrier properties and producing anti-inflammatory mediators under homeostatic conditions.^62^ In pathological settings, microglia rapidly respond to local and systemic inflammatory cues. Upon activation, they shift toward reactive phenotypes characterized by the release of cytokines such as IL-6 and TNF-α, as well as reactive nitrogen species (RNS) and ROS.^63^ These factors compromise TJs, increase BBB permeability, and perpetuate neuroinflammatory cascades. In diseases such as AD, PD, and multiple sclerosis, chronic microglial activation correlates with sustained BBB dysfunction, neurotoxicity, and cognitive decline.^64^ Systemic inflammation associated with cardiometabolic conditions can prime microglia, leading to exaggerated CNS immune responses.^65^ Circulating inflammatory mediators cross a compromised BBB and activate microglia, contributing to disease progression and neurovascular impairment.^57^ In ischemic stroke, microglia are among the earliest responders, coordinating both injurious and reparative processes depending on disease stage. Early activation promotes BBB breakdown and neuronal injury, while later responses may support tissue repair and resolution of inflammation.^66^ The ability of microglia to adapt their molecular profile and functional role in response to diverse stimuli underscores their central position in the regulation of BBB integrity and CNS immune surveillance.^67^

This intricate cellular interplay of BBB constituents is critical for the development and progression of neurological trajectories of a variety of different diseases, where sustained BBB impairment drives neuroinflammation, oxidative stress, and lastly often also neurodegeneration.

Although the BBB is tightly regulated to maintain CNS homeostasis, it is important to recognize that peripheral immune signals can communicate with both the BBB and the CNS. Activation of the peripheral immune system transmits signals to the CNS through humoral, neural and cellular pathways.^68,69^ Circulating cytokines (e.g. TNF-a, IL-1b, IL-6) signal directly to brain endothelial cells, altering tight junction proteins and glycocalyx integrity.^70,71^ Even without overt BBB disruption, these inflammatory signals can induce paracrine mediators (e.g. prostaglandins, nitric oxide, adhesion molecules like ICAM-1, VCAM-1), which activate perivascular macrophages and microglia.^68^ Under physiological conditions, immune surveillance primarily occurs in meningeal and perivascular spaces. In pathological states, such as stroke and neurodegeneration, this process is amplified, promoting microglial activation and local cytokine release.^71^ Additionally, gut-derived immune signals, shaped by microbial metabolites and dysbiosis, can further modulate systemic cytokines, BBB integrity, and CNS immune tone.^72,73^

Consequently, systemic inflammation can initiate a signaling cascade characterized by cytokine-driven activation of BBB components.^68^ Endothelial cells, pericytes, perivascular macrophages, astrocytes, and microglia detect peripheral signals, leading to localized CNS inflammatory responses.^68,71^ This tightly links systemic inflammation to neurovascular and neuroimmune dynamics.^68,71^

## Access routes of PM to the blood–brain interface

Particulate matter, particularly PM_2.5_ and PM_0.1_, can reach the brain through several converging pathways that ultimately impact the blood–brain interface. One major entry route is the olfactory pathway, where inhaled particles are deposited on the olfactory mucosa and transported retrograde along the axons of olfactory sensory neurons into the olfactory bulb.^13^ Experimental evidence demonstrates that metallic and organic PM components accumulate not only in the olfactory bulb but also in downstream regions such as the frontal cortex, hippocampus, cerebellum, and brainstem.^12,13,74^ A parallel neural route may involve the trigeminal nerve, which innervates nasal and facial structures and projects centrally into the brainstem.^74^ Though less studied, this pathway has been implicated in the translocation of aerosols in rodent models.

Beyond these neural routes, PM can access the brain indirectly via the circulatory system. Following deposition in the lungs or the gastrointestinal tract,^13,14,74^ inhaled or ingested particles may translocate across epithelial barriers^75^ or induce local inflammation that propagates systemic inflammatory signaling.^76^ Owing to their high surface area and lipophilicity, ultrafine particles are particularly capable of crossing the alveolar-capillary interface.^8,14^ Once within the blood stream, PM or its soluble constituents, including transition metals (Fe, Cu, Ni, Mn, Zn), sulfates, nitrates, and polycyclic aromatic hydrocarbons (PAHs), can impair endothelial function and promote BBB disruption.^77–80^ Transition metals catalyze Fenton-like reactions that generate ROS, whereas soluble sulfates enhance metal bioavailability and redox cycling. Organic fractions, including PAHs and nitro-PAHs, activate the aryl hydrocarbon receptor and nuclear Factor kappa-light-chain-enhancer of activated B cells (NF-κB) pathways, thereby driving pro-inflammatory cytokine release and microglial activation. Similarly, combustion-derived particles carry a diverse array of surface-bound chemicals, including transition metals and organic hydrocarbons, which render them potent inducers of inflammatory and oxidative stress responses.^81^

In parallel, ingested PM may compromise gut barrier integrity and alter microbiota composition,^82^ promoting systemic inflammation via the gut-brain axis. Specifically, gut microbiota composition shapes neuroinflammation through immune, metabolic, and barrier-related mechanisms.^83^ Dysbiosis activates pattern-recognition receptors such as toll-like receptor-4 (TLR-4), triggering MyD88–NF-κB signaling and systemic cytokine release, which primes microglia and astrocytes.^84^ Microbial metabolites regulate microglial maturation and inflammatory gene responses, support tight-junction expression, and help maintain BBB integrity.^73^ Loss of short-chain fatty acid-producing taxa promotes microglial hyperreactivity and increases BBB vulnerability.^84^ Other metabolites, including tryptophan-derived indoles and secondary bile acids, further modulate glial activation states.^85^ Dysbiosis also skews peripheral immunity, elevating circulating cytokines that act on brain endothelial cells and perivascular macrophages.^85^ Increased gut permeability and endotoxemia allow inflammatory mediators to impair the BBB, amplifying neuroinflammatory signaling.^86^ Additionally, altered microbial cues influence vagal pathways and alpha-synuclein propagation from the gut, contributing to neuroimmune activation and neurodegenerative processes.^85^

PM_2.5_ can enter the body through these three main pathways, and these routes collectively initiate convergent systemic disturbances, primarily inflammation and oxidative stress. Through the respiratory system,^87^ PM_2.5_ triggers pulmonary inflammatory signaling, which can spill into the bloodstream,^88–90^ thereby compromising BBB integrity.^87^ Gut microbiota alterations disrupt endocrine and metabolic homeostasis,^91^ leading to systemic inflammation that further affects the BBB. Finally, via the olfactory pathway,^92^ PM_2.5_ induces neuronal damage, apoptosis,^93^ and microglial activation,^94^ contributing to widespread inflammatory and oxidative cascades that propagate to the BBB.

The efficiency of PM translocation and its neurotoxic potential are further influenced by its physicochemical properties, including size, solubility, surface charge, and chemical composition.^95^ Complex mixtures that are enriched in transition metals, black carbon, and sulfates exhibit heightened toxicity.^96^ Soluble components, such as metals, ammonium sulfate, ammonium nitrate, and quinone-rich organics, are strongly associated with ROS formation, endothelial dysfunction,^97^ and neuroinflammation. Insoluble components, such as nickel and aluminum, also trigger oxidative and inflammatory responses, whereas sulphates increase metal bioavailability of redox-active metals.^98^ Lipophilic organic compounds can cross cellular membranes and interact directly with the CNS.

Host factors significantly modulate vulnerability to PM-induced neurotoxicity. Aging is accompanied by microglial priming, impaired antioxidant capacity, and increased BBB permeability, increasing the sensitivity to PM-induced insults exposure. Sex-related differences in immune signaling and xenobiotic metabolism may further shape susceptibility profiles. Metabolic or cardiovascular comorbidities, including hypertension and diabetes, amplify systemic inflammation, and potentiate oxidative cascades initiated by PM. Socioeconomic determinants, which influence both exposure burden and baseline health status, additionally contribute to population-level disparities, placing children, older adults, and individuals with chronic disease at elevated risk.^11^

ROS are a key mechanistic link between these access routes and neurovascular injury^64,99^ (Table 1). PM_2.5_ and ultrafine particles promote ROS formation directly, via transition metals, quinones, and organic radicals on their surfaces, and indirectly, by activating endogenous oxidase systems, such as Nicotinamide Adenine Dinucleotide Phosphate (NADPH) oxidase^100^ (NOX2 and NOX4^101^), xanthine oxidase, and mitochondrial electron transport chain complexes. Superoxide (O_2_^−^·), hydrogen peroxide (H_2_O_2_), and hydroxyl radicals (·OH) are among the most relevant ROS species and play central roles in neurovascular pathology.^97,102–105^ In cerebral endothelial cells, O_2_^−^· and H_2_O_2_ destabilize TJs by oxidatively modifying occludin and zona occludens (ZO-1) and by engaging redox-sensitive kinases, including protein kinase C, protein kinase Src, and mitogen-activated protein kinases (MAPKs). Astrocytes^106^ respond to PM-induced oxidative stress with increased mitochondrial ROS production, NF-κB activation, and release of IL-6 and TNF-α, thereby propagating inflammatory signaling at the BBB. Microglia^103^ generate both O_2_^−^· and peroxynitrite (ONOO^−^) through coordinated activation of NADPH oxidase and inducible nitric oxide synthase (iNOS), contributing to neuronal and endothelial injury. Although less comprehensively studied, pericytes also exhibit elevations in mitochondrial H_2_O_2_ and NOX4-derived O_2_^−^·, promoting pro-inflammatory and contractile phenotypes. Together, these ROS-driven processes^107^ disrupt endothelial integrity, increase BBB permeability, and amplify neuroinflammatory cascades within the NVU.

**Table 1.** Key ROS generated by PM exposure and their effects on NVU cell types and pathways. ROS listed in the table are linked to their predominant sources and the specific NVU targets they modulate during PM exposure.

ROS species	Major sources (in NVU cells)	Primary molecular targets	Cell types affected	Main effects
O_2_^−^·	NADPH oxidase (NOX2^108^/NOX4), mitochondria	Lipid peroxidation, activation of MAPKs and PKC, TJ protein oxidation	Endothelial cells,^109^ microglia,^100^ pericytes	TJ disruption, microglial activation,^110^ vascular tone alteration^111^
H_2_O_2_	Dismutation of O_2_^−^·, mitochondria	NF-κB activation,^112^ oxidative modification of ZO-1/claudin-5	Endothelial cells,^104^ microglia,^113^ astrocytes,^114,115^ pericytes	Inflammatory signaling, endothelial barrier breakdown
·OH	Fenton reaction (Fe^2+^ + H_2_O_2_)	DNA strand breaks, lipid and protein oxidation	Neurons, endothelial cells	Cell death, necrosis/apoptosis
ONOO^−^	Reaction of O_2_^−^· with NO· (from iNOS)	Nitration of tyrosine residues, mitochondrial dysfunction^116^	Microglia, neurons, endothelial cells^103^	Neurotoxicity, mitochondrial failure
^1^O_2_	PM metal-organic reactions, photochemical oxidation	Protein oxidation, membrane damage	Endothelial cells	Oxidative stress, reduced cell viability

DNA: desoxyribonucleic acid; Fe: iron; MAPK: mitogen-activated protein kinase; NF-κB: nuclear factor kappa-light-chain-enhancer of activated B cells; NOX: NADPH oxidase; PM: particulate matter; NVU: neurovascular unit; ROS: reactive oxygen species; TJ: tight junction; ZO-1: zona occludens 1; O_2_^−^·: superoxide anion; H_2_O_2_: hydrogen peroxide; ·OH: hydroxyl radical; ONOO^−^: peroxynitrite; ^1^O_2_: singlet oxygen.

The combination of diverse access pathways with potent ROS-mediated injury highlights the multifactorial nature of PM’s impact on the neurovascular system and underscores the complex interplay between environmental exposure and neurovascular health.^95^

## Endothelial targets of PM_2.5_ with consequences for BBB integrity

Exposure to PM has been demonstrated to induce significant endothelial dysfunction,^79^ with oxidative stress representing a primary contributing factor.^117^ PM_2.5_ components including transition metals and organic compounds enhance ROS production via NOX activation,^118^ initiating cellular damage and disruption of endothelial function.^111,118^ Specifically, PM_2.5_ has been shown to increase levels of oxidized phospholipids in the lungs, which propagate a systemic inflammatory response through the TLR4/NOX-dependent signaling pathway,^111^ thereby contributing to systemic endothelial dysfunction, including at the BBB. This oxidative burden impairs NO bioavailability, leading to endothelial activation and reduced vasodilatory capacity.^119^ Pharmacological inhibition of NOX1/4 has been shown to mitigate these adverse effects.^118^ In addition, ROS-mediated endothelial barrier disruption was linked to altered calcium influx via transient receptor potential (TRP)-M2 channels and subsequent calpain activation.^78^ Moreover, PM_2.5_-induced ROS cause endothelial membrane damage and increase permeability through activation of VEGF receptor 2 (VEGFR2) and MAPK/extracellular signal-regulated kinases (ERK) pathways, leading to tissue inflammation and endothelial dysfunction.^120^

Oxidative stress and inflammation are tightly interconnected,^121^ and the pro-oxidant effect of PM_2.5_ also serves as trigger for inflammatory cascades in endothelial cells.^122^ In particular, the NLR family pyrin domain containing 3 (NLRP3) inflammasome has emerged as a critical mediator of PM_2.5_-induced endothelial dysfunction, with ROS acting as an important upstream activator.^123^ Likewise, PAH, significant constituents of PM_2.5_, activate oxidative stress and inflammatory pathways including nuclear factor erythroid 2-related factor 2 (Nrf2)/heme oxygenase-1 (HO-1) and NF-κB/TNF-α,^124^ and alter endothelial cell function via cyclooxygenase 2 (COX-2)/prostaglandin E2 (PGE2) and ERK/protein kinase B (AKT)/NF-κB signaling.^77^ Together, these mechanisms promote vascular injury^125^ and have been implicated in age-related vascular pathologies.^126^ Therapeutic strategies targeting this axis, such as the use of antioxidants like N-acetylcysteine (NAC) or NLRP3 inhibitors, have been shown to attenuate PM_2.5_-induced endothelial damage.^123^

Together with inflammatory and ROS responses, the function of endothelial cell surface molecules is essential for maintaining BBB integrity. PM_2.5_ exposure leads to upregulation of adhesion molecules such as ICAM-1 and VCAM-1,^127,128^ which facilitate leukocyte adhesion and transmigration and hence, exacerbates vascular inflammation.^129^ Both ICAM-1 and VCAM-1 are upregulated via NF-κB activation,^128^ a key inflammatory signaling pathway also responsible for the expression of pro-inflammatory cytokines like IL-6 and TNF-α.^130,131^ Activation of these cytokines further contributes to BBB disruption through the degradation of TJ proteins, including occludin and claudin-5.^132–134^ Tight and adherens junctions are essential to BBB integrity^15^ and are significantly affected by PM_2.5_. In vitro studies revealed reduced levels of TJ proteins ZO-1 and ZO-2 following PM_2.5_ exposure, with calpain-mediated degradation and ZO-1 redistribution associated with increased permeability and monocyte migration.^78,127^ Moreover, PM_2.5_-activated perivascular macrophages impair endothelial function by enhancing autophagic flux and promoting degradation of TJs.^17^ PM_2.5_ also reduces vascular endothelial (VE)-cadherin levels,^120^ thereby destabilizing endothelial contacts. This effect is partially mediated by miR-21, which is upregulated by PM_2.5_ and concurrently reduces VE-cadherin and metalloproteinase inhibitor 3 (TIMP3) expression, while promoting matrix-metalloproteinase (MMP) activation, contributing to junction degradation.^18,120,135^ Transcriptomic changes in endothelial cells exposed to PM_2.5_ reveal elevation of microRNAs such as let-7a and miR-21,^136^ both implicated in barrier dysfunction.^136^ miR-21 specifically represses TIMP3, enabling increased MMP-9 activity and leading the expression of SRY-box transcription factor-7 (SOX-7) and endothelial degradation.^18,135^ In vivo, ApoE^−/−^ mouse models confirm these findings, showing increased BBB permeability, ROS production, and MMP-2/-9 activity after PM exposure.^137^ The miR-21 pathway activated after PM_2.5_ exposure also regulates the NLRP3 inflammasome via TLR4/NF-κB signaling.^138^ In endothelial cells, PM_2.5_ exposure has been associated with IL-6 induction dependent on the Janus kinase 1/signal transducer and activator of transcription 3 (JAK1/STAT3) pathway,^139^ which in other models of PM exposure has been linked to altered cell proliferation through downregulation of cyclin D1 and activation of miR-21.^140^ Furthermore, miR-21 has been implicated in the regulation of the phosphatase and tensin homolog/phosphoinositide 3-kinase/protein kinase B (PTEN/PI3K/AKT) signaling pathway.^141^

PM_2.5_ exposure further exacerbates endothelial injury by activating components of the local renin-angiotensin system. In particular, it induces upregulation of the angiotensin II type 1 receptor (AT1R) through MAPK and hypoxia inducible factor 1α (HIF-1α) signaling pathways.^142^ In parallel, PM_2.5_ and metal-associated particles impair mitochondrial and lysosomal integrity, leading to reduced mitochondrial adenosine-triphosphate (ATP) production.^143^ These organelle dysfunctions promote endothelial apoptosis^144^ and further compromise the structural and functional integrity of the BBB.

Finally, PM_2.5_ disrupts endothelial crosstalk with astrocytes and pericytes by impairing the secretion of key signaling molecules, including angiopoietin-1 (Ang-1), VEGF, and transforming growth factor beta (TGF-β).^143,145,146^ For example, PM_2.5_-induced reductions in Ang-1 weaken astrocytic end-feet adhesion and destabilize endothelial TJs,^146^ thereby increasing BBB permeability.

These molecular and cellular disturbances collectively underscore the multifaceted and detrimental effect of PM_2.5_ on endothelial function and BBB integrity, establishing a mechanistic link between environmental pollution and the development of neurovascular and neurodegenerative pathologies.

## Mechanisms of PM-induced pericyte alterations

Current knowledge regarding the impact of PM exposure on pericytes remains limited, but emerging evidence suggests that pericytes are affected and may play a critical role in PM-induced neurovascular dysfunction. One study reported the accumulation of lipofuscin granules, a marker of cellular aging and stress, in pericytes and other vascular-associated cell types within the prefrontal white matter, potentially indicating compromised NVU function.^147^ Exposure to PM_2.5_ has been associated with a reduction in pericyte coverage of brain capillaries, which is thought to arise in part from impaired endothelial secretion of pericyte-recruiting signals such as platelet-derived growth factor-BB (PDGF-BB). This decline in pericyte recruitment compromises microvascular stability^148,149^ and may contribute to BBB leakage and heightened neuroinflammation. First, in vitro data using cultured human brain pericytes further support this hypothesis. Exposure to diesel exhaust particles,^150^ a major component of PM, has been shown to increase IL-6 expression and impair wound closure capacity, reflecting both a pro-inflammatory shift and functional impairment of pericytes.

Despite these emerging insights, it is important to emphasize that much of the existing evidence comes from animal studies and in vitro systems. While these models are invaluable for mechanistic investigations, they only partially represent the complexity of the human neurovascular environment, show apparent differences on the transcriptome level^151^ and diverge functionally.^152^ Currently, there is limited human data on pericyte-specific alterations following PM_2.5_ exposure, and most of the available evidence is indirect or inferred from broader vascular or inflammatory outcomes.^153,154^ Future studies employing human-relevant approaches, including advanced neuroimaging, postmortem analyses, and engineered human NVU models,^106^ such as organoids and microfluidic platforms, are needed to validate pericyte-mediated mechanisms in the human context. Closing these gaps is essential to establishing the relevance of pericyte dysfunction in air pollution-related neurovascular pathology (Supplemental References).

Given the suggested role of pericytes in maintaining NVU integrity,^43,46,155^ regulating BBB permeability, and modulating neuroinflammatory responses, there is an urgent need for more focused research into the effects of PM exposure on pericyte biology. Understanding these mechanisms may reveal new insights into how environmental pollutants contribute to CNS pathologies through NVU destabilization and will help clarify the extent to which pericyte impairment observed in experimental systems extrapolates to the human condition.

## Astrocyte responses to PM exposure: Mechanisms of BBB alterations

Astrocytes are highly sensitive to environmental insults, including exposure to PM_2.5_, which can induce their transformation into a reactive state.^145^ Multiple in vivo studies have demonstrated that PM exposure increases the expression of glial fibrillary acidic protein (GFAP),^19^ a widely recognized marker of astrogliosis. Recent data suggest that PM exposure can induce both A1 (neurotoxic, pro-inflammatory) and A2 (neuroprotective, anti-inflammatory) phenotypes in cortical astrocytes,^156^ indicating a complex and context-dependent response.^157^ In vitro experiments have shown that astrocytes are not only responsive to inflammatory cues from microglia but can also autonomously release pro-inflammatory mediators upon PM_2.5_ exposure.^156,158^ PM_2.5_ exposure of astrocytes induces oxidative stress through the generation of ROS and RNS,^158,159^ which fuels the secretion of pro-inflammatory cytokines such as IL-1β, TNF-α, and IL-6. Specifically, IL-6 activates the JAK/STAT3 pathways,^160^ enhancing the expression of adhesion molecules (e.g. ICAM-1, VCAM-1^160^) and facilitating leukocyte adhesion and transmigration across the barrier. TNF-α, through NF-κB activation, promotes MMP expression and cytoskeletal rearrangement, leading to TJ disassembly and endothelial dysfunction.^161,162^ Collectively, these processes weaken endothelial cohesion and promote leukocyte infiltration and neuroinflammation.

Although transient astrocyte activation may represent a protective mechanism, sustained oxidative and inflammatory stress can impair the finely tuned crosstalk astrocytes maintain with other CNS cell types.^163^ Disruption of astrocyte-microglia communication may amplify inflammatory responses, while impaired glutamate clearance at tripartite synapses can result in excitotoxicity and progressive neuronal injury.^164,165^ This loss of homeostatic function is particularly concerning given the close association between astrocytes and the cerebral vasculature: nearly all cortical astrocytes contact at least one blood vessel via their perivascular end-feet.^166^ These structures play a crucial role in both the formation and maintenance of the BBB.^167,168^

Astrocytes can influence BBB integrity through secretion of vascular permeability factors such as VEGF, NO, and MMPs, as well as protective molecules like Ang-1 and glial cell line-derived neurotrophic factor (GDNF).^169^ PM exposure may dysregulate this balance, leading to BBB compromise through mechanisms that remain incompletely understood. While direct astrocyte-mediated BBB disruption following PM exposure has not yet been conclusively demonstrated in vivo, several studies implicate altered astrocyte-endothelial signaling in this process.^137^ Specifically, PM_2.5_-induced inflammatory signaling affects key pathways, including VEGF and TGF-β, which are critical for BBB stability. VEGF upregulation increases vascular permeability by downregulating TJ proteins, such as claudin-5 and occludin and by inducing endothelial fenestrations via VEGFR2-mediated phosphorylation cascades.^170^ In contrast, aberrant TGF-β signaling, particularly chronic activation, can impair endothelial differentiation and promote extracellular matrix remodeling that destabilizes the basement membrane.^171^

Pro-inflammatory cytokines such as IL-6 and TNF-α, which are upregulated in response to PM, further promote astrocyte reactivity and may impair neurovascular coupling.^172^ These mechanisms are particularly relevant in the context of CNS diseases such as ischemic stroke, where PM exposure could exacerbate disease onset or severity by impairing BBB function and amplifying neuroinflammation.^173^ Support for these mechanisms comes from a recent study utilizing a 3D human brain-on-a-chip microfluidic model, in which astrocytes proliferated in response to PM_2.5_ and secreted elevated levels of chemokines (e.g. C–C motif ligand-1 (CCL-1), CCL-2) and cytokines (e.g. IL-1β, interferon-γ (IFN-γ)).^106^ These astrocyte-derived mediators were shown to recruit and activate microglia, which subsequently released additional pro-inflammatory cytokines and NO, exacerbating neuronal damage.^106^ Such in vitro multicellular models are increasingly valuable for dissecting the complex cellular interactions at the BBB, which are often difficult to resolve using in vivo approaches alone.

Moreover, astrocytes play a central role in glymphatic function, with AQP4 being essential for water clearance.^29^ PM_2.5_ exposure upregulates AQP4 in astrocytes^174,175^ and enlarges perivascular spaces, correlating with impaired glymphatic clearance and cognitive decline.^176^ Combined PM-induced neuroinflammation, peripheral inflammation, and endothelial damage further reduce glymphatic efficiency, promoting protein aggregation associated with neurodegeneration and dementia.^177,178^ These indirect effects of PM on the glymphatic system remain poorly understood and require further investigation, including non-invasive approaches.^179^

Together, these findings suggest that astrocytes may play an important modulatory role in PM-induced neuroinflammation and BBB dysfunction, although their precise contribution remains to be fully elucidated.

## Mechanisms underlying PM-induced microglia activation: Implications for BBB integrity

Microglia, the resident immune cells of the brain, respond to PM_2.5_ and PM_0.1_ exposure with activation, characterized by morphological changes and upregulation of pro-inflammatory markers such as IL-1β, TNF-α, and IL-6,^180^ as well as increased production of ROS and NO.^145,181^ This reactive microglial phenotype is further supported by elevated expression of inflammatory genes like COX-2 and inducible nitric oxide synthase (iNOS) in brain regions such as the hippocampus,^181,182^ alongside reductions in neurotrophic factors like brain derived neurotrophic factor (BDNF).^183^ Diesel exhaust particles similarly elevate CCL-3, fractalkine, ionized calcium-binding adapter molecule 1 (Iba-1), and oxidative stress markers like malondialdehyde.^103^ These factors contribute to caspase-1 activation and apoptosis,^180,181^ mediated in part by NF-κB phosphorylation when Nrf2 is suppressed,^184^ further exacerbating neuroinflammatory responses.^121^ This inflammatory milieu can contribute to BBB disruption, promoting TJ disassembly and facilitating immune cell infiltration.^185^ Together, these alterations predispose to the development of structural brain alterations and cognitive deficits.^147^

Mechanistically, PM_2.5_ promotes microglial polarization toward reactive phenotypes,^107^ a shift, that is, driven by oxidative stress pathways and exacerbated by inflammatory cytokines such as IFN-γ and IL-1β.^186^ This is reflected in the upregulation of microglia markers such as cluster of differentiation (CD) 86, CD11b, and iNOS, as well as increased expression of CD68.^106,187^ Simultaneously, PM_2.5_ appears to inhibit polarization towards anti-inflammatory and reparative phenotypes, in part through suppression of mechanistic target of rapamycin (mTOR) signaling.^106^ Markers characteristic of the microglia with an anti-inflammatory phenotype, including CD206, IL-10, TGF-β, insulin-like growth factor (IGF-1), nerve growth factor (NGF), and BDNF, are not upregulated following PM exposure,^106^ suggesting a loss of neuroprotective function. Since microglial cells can regulate BBB integrity through context-dependent activation states, where reactive microglial contribute to barrier disruption and leakage, while microglia with homeostatic or reparative phenotypes support barrier maintenance,^188^ this regulatory loop between endothelial cells and microglia is likely disrupted by PM_2.5_ exposure, with significant implications for BBB structural integrity.

At the organelle level, PM_2.5_ exposure leads to mitochondrial dysfunction in microglia, characterized by membrane depolarization, increased energy demand, reduced ATP, and elevated reduced form of nicotinamide adenine dinucleotide (NADH),^189,190^ which are indicators of a metabolic shift toward glycolysis and oxidative stress.^181,191^ These bioenergetic disturbances are closely tied to the activation of the NLRP3 inflammasome via the high mobility group box 1 (HMGB1)–P2X7 purinergic receptor axis, resulting in elevated IL-1β and IL-18 and contributing to synaptic damage and neuronal apoptosis.^192,193^ Moreover, microglial P2X7 receptor is likely to play a central role both in sensing extracellular ATP released during stress and in amplifying NLRP3-driven inflammation.^192^ ATP as an important mediator for neuronal response to extracellular disturbances and a signaling molecule for the neuron-microglia crosstalk^194^ is largely affected by concentrated ambient particles,^195^ including PM that induce a decay in the levels of intracellular ATP and depolarized mitochondrial membranes.^190^

Although the above effects of PM_2.5_ on microglia are well-documented, it is important to consider the spatial-temporal dimension of this process. In many instances, augmented microglial reactivity may be a secondary response, occurring after PM_2.5_-induced compromise of the BBB.^196^ Conversely, microglia also actively regulate BBB integrity. Reactive microglia exacerbate BBB dysfunction through cytokine release and matrix degradation, whereas the polarization towards reparative microglia phenotypes supports endothelial integrity. This bidirectional crosstalk forms a regulatory loop, wherein PM_2.5_-induced microglia polarization contributes to a cycle of BBB breakdown and sustained neuroinflammation.^107^

Microglial activation in this context is also associated with altered communication between microglia and astrocytes.^145^ Activated microglia release IL-1β and TNF-α, inducing astrocytic reactivity and promoting chemokine secretion (e.g. CCL2), further amplifying inflammation.^197^ Additionally, microglia-derived extracellular vesicles (EVs), enriched with inflammatory proteins such as Alix and flotillin-2,^184^ can disrupt astrocytic support of endothelial cells and increase BBB permeability. One notable EV cargo is glutaminase C,^184^ which contributes to glutamate excitotoxicity and decreased neuronal viability,^127,184^ a process that can be partially mitigated by non-N-methyl-D-aspartate (NMDA) receptor antagonists.^127^ Disruption of microglia-astrocyte communication^163^ by pollutants such as PM_2.5_ may contribute to the loss of homeostasis^198^ and promote neuronal dysfunction through mechanisms like glutamate excitotoxicity and synaptic degradation,^199^ resembling pathological processes observed in neurodegenerative diseases and stroke.^199,200^

Finally, PM_2.5_ exposure can transiently upregulate disease-associated microglial markers such as triggering receptor expressed on myeloid cells 2 (TREM2) and lipoprotein lipase (LPL),^181,201^ suggesting some neuroprotective attempt. However, this response appears limited and is eventually overshadowed by a more robust and persistent phenotype with pro-inflammatory features.^106^ The resulting inflammatory environment, glutamate-mediated excitotoxicity, and impaired neurovascular signaling contribute to key pathological features of both neurodegenerative and cerebrovascular diseases.^202,203^

Together, PM_2.5_-induced microglia-driven inflammation may link to synaptic dysfunction and neuronal apoptosis, which are critical features in classical neurodegenerative diseases.^204^ In cerebrovascular diseases,^205^ reactive microglia additionally release MMPs, further degrading the BBB and facilitating leukocyte infiltration into the brain parenchyma,^206^ highlighting their possible contribution to BBB degeneration once primed toward pro-inflammatory phenotypes.

## PM-induced alterations in BBB transporter and enzyme function

Beyond their well-described effects on TJ integrity, airborne PM can also disrupt the biochemical barrier functions of the BBB, which depend on the coordinated activity of transporter systems and metabolic enzymes. These components critically regulate selective permeability and support CNS homeostasis.^207^ Growing evidence indicates that PM exposure alters the expression and function of several BBB efflux transporters, particularly under inflammatory or oxidative conditions. Diesel exhaust particles and mixed vehicle emissions have been shown to reduce P-glycoprotein (P-gp/ABCB1) expression and activity in BBB models co-cultured with microglia, leading to increased paracellular permeability and reduced efflux capacity.^110^ In vivo in mice exposed to vehicle emissions^137^ show similar P-gp suppression. Similarly, inflammatory cytokines and oxidative stress, both prominent responses to PM exposure,^106^ have been associated with the impairment of breast cancer resistance protein (BCRP) and multidrug resistance-associated protein (MRP) transporter activity in endothelial cells,^208^ suggesting that PM-induced neuroinflammation may diminish efflux capacity at the BBB.

Alterations in uptake transporters have been less studied in the context of PM exposure. While inflammation reduces L-type amino acid transporter 1 (LAT1) expression^209^ at the BBB in rodents, and PM exposure downregulates glucose transporter type 1 (GLUT1)^210^ and other nutrient transporters in the placenta and pulmonary endothelium, direct evidence for such effects at the BBB is currently lacking. These observations from peripheral tissues may, however, be mechanistically relevant, as similar oxidative and inflammatory pathways operate in brain microvascular endothelium. This represents a critical knowledge gap, as reduced GLUT1 or LAT1 expression could impair cerebral energy and amino acid supply even in the absence of overt structural barrier disruption.

PM exposure may also influence xenobiotic-metabolizing enzymes, including CYP1A1 and CYP2E1,^211–214^ in BBB and NVU cells, although much of the available evidence derives from systemic, lung, or hepatic models. Given the presence of such enzymes within BBB endothelium, altered metabolic activity could further modulate xenobiotic handling under PM-induced stress.

In addition to transporters and metabolic enzymes, PM can affect proteolytic enzyme systems that regulate BBB structure. PM exposure upregulates MMPs, particularly MMP-2 and MMP-9,^137^ via oxidative and inflammatory signaling pathways. Although MMPs are not metabolic enzymes, their proteolytic activity contributes to BBB dysfunction by degrading components of the basal lamina and TJ-associated scaffolding. Thus, PM-induced activation of MMPs represents an additional enzymatic mechanism by which barrier integrity may be compromised.

Collectively, these observations indicate that PM-induced BBB dysfunction involves not only structural impairment of TJs but also biochemical alterations in transporter, metabolic, and proteolytic enzyme systems. Although direct in vivo evidence at the BBB remains limited, emerging data highlight transporter and enzyme dysregulation as underrecognized mechanisms linking PM exposure to neurovascular and neurodegenerative outcomes.

## Clinical relevance of PM_2.5_ effects

Human and population-level studies provide strong translational support for the experimental findings described in this review. A broad body of evidence indicates that chronic PM_2.5_ exposure induces systemic inflammation and immune dysregulation across diverse populations.^215–217^ Large multi-ethnic cohorts have shown that exposure to PM_2.5_ is associated with elevated inflammatory and hemostatic markers, including increased high-sensitivity C-reactive protein (hs-CRP), white blood cell counts, platelet numbers, fibrinogen levels, and circulating cytokines such as IL-6, IL-1β, and TNF-α.^218,219^ Additional studies report increases in endothelial microparticles, sICAM-1, and sVCAM-1,^220^ as well as upregulation of VEGF and other mediators linked to vascular inflammation.^221^ Many of these effects are amplified in individuals living near high-traffic areas^216^ and in vulnerable patient groups such as those with pre-diabetes or ischemic heart disease,^222,223^ suggesting that PM-induced oxidative stress and chronic systemic inflammation may exacerbate cardiovascular and autonomic dysregulation. In relation to cardiovascular diseases, PM_2.5_ has been associated with increase in cardiovascular diseases risk. Cohort studies over the world and meta-analysis have reported that chronic or long-term exposure to PM_2.5_ resulted in an increased cardiovascular risk by increase of blood pressure and hypertension,^224,225^ and increase hospitalization rate, risk and incidence of myocardial infraction,^226,227^ ischemic stroke,^228–230^ heart failure,^231,232^ and arrhythmia.^233,234^

Transcriptomic studies further underscore the widespread immunological impact of PM_2.5_ exposure in humans. Recent cohorts from the Netherlands and Colombia show PM-associated alterations in the expression of genes involved in inflammation, IFN signaling, apoptosis, mitochondrial electron transport, platelet activation, and immune-cell recruitment.^153,235,236^ Upregulation of chemokines linked to neutrophil, monocyte, and natural killer-cell trafficking, activation of IL-36–mediated pathways,^235^ and reduced expression of RNases and pathogen-response genes collectively point to a state of immune dysregulation that may impair host defense while promoting chronic inflammation.

These systemic responses are paralleled by evidence of direct effects on the human brain. Post-mortem analyses reveal that individuals with higher lifetime exposure to traffic-related PM_2.5_ exhibit more severe AD neuropathology, including increased amyloid plaque burden, higher CERAD scores, and markers of microglial activation and oxidative stress in the brain.^237^ Other human studies indicate that exposure to PM_2.5_ is associated with elevated biomarkers related to AD, including structural MRI alterations, changes in cerebrospinal fluid markers, and amyloid positron emission tomography signals.^238–243^ These neuropathological findings parallel the neuroinflammatory and barrier dysfunction mechanisms observed in preclinical models, suggesting that PM_2.5_ exposure contributes directly to neurodegenerative processes.

Apart from AD, experimental and epidemiological evidence supports a link between exposure to PM_2.5_ and PD. Several studies show that higher ambient PM_2.5_ exposure increases the risk of PD onset and may influence disease phenotype and progression. Long-term PM_2.5_ exposure is associated with an increased pooled risk,^244^ while a recent updated meta-analysis reported a smaller but still statistically significant hazard ratio of PM_2.5_ exposure contributing to PD risk.^245^ A large population-based cohort study in Canada^246^ and Northern Ireland^247^ reported significant increases in PD incidence in PM_2.5_ exposure. For healthcare systems, the hospitalization rate of patients is also relevant, in case of PD patients this rate has been linked to short-term PM_2.5_ exposure.^248^ Emerging clinical evidence suggests that PM_2.5_ may also shape the clinical phenotype and severity. Higher exposure to PM_2.5_ is associated with increased risk of developing dyskinesia and the akinetic-rigid phenotype compared to PD patients exposed to lower levels.^249^ Experimental studies showed that PM_2.5_ promotes fibrilization and aggregation of alpha-synuclein and exacerbates dopaminergic neuronal degeneration.^250^ Further processes central to PD pathogenesis are reinforcing the population observation in experimental models. PM_2.5_- associated ROS production increases mitochondrial dysfunction, reduced autophagy/mitophagy (reduced microtubule-associated protein 1 light chain 3-II (LC3II), autophagy-related protein 5, (Atg5); increased mTOR), and increased inflammatory cytokines (TNF-α, IL-1β, IL-6) in the substantia nigra.^251^ PM_2.5_-triggered systemic inflammation can propagate to the brain and induce the release of inflammatory factors, promote neuroinflammation and contribute to the loss of dopaminergic neurons and alpha-synuclein accumulation.^252,253^ Other possible mechanistic links are the possibility of PM_2.5_ to cross and disrupt the BBB, impair endothelial function and promote vascular inflammation, however these are less studied and warrant further investigation.

Large epidemiological cohorts reinforce this translational connection. Prospective analyses from the UK Biobank and other population studies link long-term PM_2.5_ exposure to an increased risk of all-cause dementia, including AD and emerging evidence for Lewy-body dementia.^254^ Longitudinal cohorts also report associations between higher PM_2.5_ and accelerated cognitive decline, even at exposure levels below many current regulatory standards, emphasizing that seemingly “low” ambient levels may still pose a significant risk to brain health.^255^ Meta-analyses reinforce these findings, demonstrating consistent associations between chronic PM exposure and age-related neurodegenerative outcomes across diverse populations, including AD, PD, vascular dementia, amyotrophic lateral sclerosis, and cognitive function decline.^256^ Recent advances in human 3D brain-on-chip models provide additional mechanistic validation, demonstrating that BBB-penetrating PM_2.5_ can directly induce astrogliosis, reactive microgliosis, and downstream neuronal injury, including synaptic impairment and tau phosphorylation.^106^ These findings support that PM_2.5_ exposure contributes to the activation of different pathways known to drive neurodegeneration and leading to an increase of AD and PD risk and influencing disease progression. Together, these experimental and human findings support the concept that PM_2.5_ acts as a modifiable environmental risk factor for dementia and related neurodegenerative disorders.

Understanding the effects of PM_2.5_ requires integrating clinical studies with in vivo and advanced in vitro models.^257^ Emerging 3D BBB systems and imaging tools provide valuable insights into PM_2.5_ interactions with the brain.^258^ PM_2.5_ crosses 3D BBB organotypic chip, inducing cytotoxicity and astrocyte proliferation via oxidative stress, which is consistent with in vivo mouse studies that show BBB disruption, increased permeability, endothelial cytotoxicity, and neuroinflammation.^115^ Mice also exhibit elevated GFAP-positive cells,^259,260^ mirroring patterns observed in children,^261^ and postmortem AD.^262^ Imaging approaches, including ex vivo MRI, revealed structural and diffusivity changes in rat brains after PM exposure,^263^ while PET imaging with labeled PM_2.5_ confirmed brain entry, disturbed glucose metabolism, and persistent neuroinflammation months after PM exposure.^264^ Particle accumulation is sustained, with increased uptake in multiple brain regions.^264^ Although 2-photon microscopy is widely used to study BBB function, its application to PM_2.5_ has largely focused on pulmonary deposition.^265^ Together, non-invasive imaging techniques and state-of-the-art in vitro models promise to advance our understanding of PM2.5’s impact on brain health.

Collectively, the integration of mechanistic, clinical, and epidemiological evidence underscores the urgency of identifying PM components most responsible for neurotoxicity, developing early biomarkers of exposure-related brain injury, and devising strategies to protect vulnerable populations from environmentally driven neurodegenerative processes.

## Conclusion

**Figure 1.**
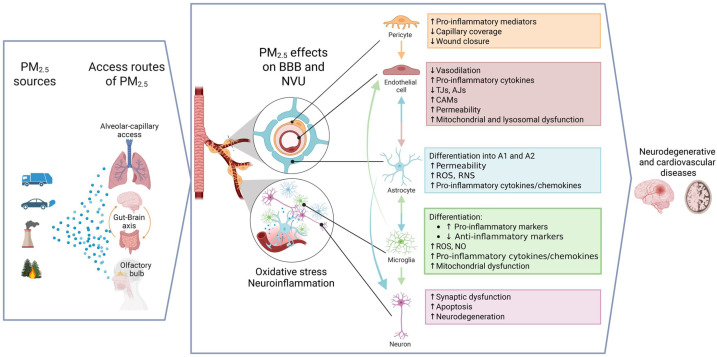
Graphical summary of key PM-induced effects on the BBB and neurovascular cell interactions. Overview illustrating how PM, particularly PM_2.5_, enters the body and initiates systemic and central responses. The figure summarizes the principal signaling pathways activated by PM exposure, the resulting oxidative and inflammatory processes, and the subsequent interactions among key cell types within the neurovascular unit, including endothelial cells, astrocytes, microglia, and perivascular macrophages. Together, these pathways and cell-to-cell communications depict the current understanding of how PM exposure contributes to BBB dysfunction and neuroinflammation. Image generated with BioRender. PM: particulate matter.

In conclusion, PM_2.5_ exposure profoundly compromises BBB integrity through a cascade involving oxidative stress, inflammatory signaling, and dysregulation of endothelial interactions with astrocytes and pericytes. These insults collectively disrupt endothelial TJs, impair astrocytic metabolic and trophic support, polarize microglia toward pro-inflammatory phenotypes, and suppress endogenous neuroprotective responses. BBB dysfunction further promotes infiltration of peripheral immune cells and the release of extracellular vesicles enriched in neurotoxic mediators such as glutamate, cytokines, and matrix-degrading enzymes, amplifying neuronal injury (Figure 1).

Despite substantial progress, critical gaps remain in our understanding of the spatiotemporal dynamics of PM_2.5_-induced BBB disruption. In particular, the specific signaling mechanisms by which PM_2.5_ perturbs endothelial homeostasis, pericyte contractility and survival, and astrocyte-endothelial crosstalk are poorly defined. The roles of microglia-derived inflammatory mediators and extracellular vesicles in modulating BBB permeability and endothelial metabolism also remain incompletely characterized. Moreover, how chronic, low-dose PM_2.5_ exposure interacts with systemic vascular or metabolic comorbidities (e.g. hypertension, diabetes, dyslipidemia) to exacerbate neurovascular dysfunction is still largely unexplored. Finally, the relative contributions of paracellular versus transcellular transport alterations, including dysregulation of endothelial transporters, enzymes, and lipid signaling pathways, warrant systematic investigation using advanced in vitro and in vivo models.

Future research should prioritize integrated in vivo and organ-on-chip models that capture the multicellular complexity of the NVU under realistic exposure conditions, enabling delineation of causal relationships and dose thresholds. Therapeutically, promising directions include targeting redox imbalance (e.g. Nrf2 activators, mitochondrial antioxidants), modulating inflammatory signaling (e.g. TLR4/NF-κB inhibitors, cytokine blockade), and restoring BBB integrity via stabilization of TJs, pericyte–endothelial signaling, or astrocyte–endothelial signaling. Ultimately, combining preventive environmental policies with mechanism-based pharmacological or nutritional interventions offers the most effective path to mitigate the long-term neurological burden of air pollution.

## Supplemental Material

sj-docx-1-jcb-10.1177_0271678X261418925 – Supplemental material for Airborne particulates and brain health: The role of PM2.5 in blood–brain-barrier dysfunction Supplemental material, sj-docx-1-jcb-10.1177_0271678X261418925 for Airborne particulates and brain health: The role of PM2.5 in blood–brain-barrier dysfunction by Fátima Gimeno-Ferrer, Lisa Teresa Porschen, Frank Matthes, Katrin Gohlsch and Anja Meissner in Journal of Cerebral Blood Flow & Metabolism
