# Supplementary material for: Airborne particulates and brain health: The role of PM2.5 in blood–brain-barrier dysfunction
Source: J Cereb Blood Flow Metab. 2026 Feb 8:0271678X261418925. Online ahead of print. doi: 10.1177/0271678X261418925 (PMC12885965; doi:10.1177/0271678X261418925)
Supplement: sj-docx-1-jcb-10.1177_0271678X261418925 – Supplemental material for Airborne particulates and brain health: The role of PM2.5 in blood–brain-barrier dysfunction [file sj-docx-1-jcb-10.1177_0271678X261418925.docx]

**Main text References cont.**

151. Miao Y, Li W, Jeansson M et al. Different gene expression patterns between mouse and human brain pericytes revealed by single-cell/nucleus RNA sequencing. *Vasc Pharmacol* 2024; 157**:** 107434.

152. Stevenson TJ, Lee K, Li S et al. Heterogeneity in pericyte inflammatory responses across age and species highlight the importance of human cell models. *Mol Brain* 2025; 18(1)**:** 37.

153. Rundblad A, Das S, Ginos BNR et al. Exposure to fine particulate matter in adults is associated with immune cell gene expression related to inflammation, the electron transport chain, and cell cycle regulation. *Environ Epigenet* 2025; 11(1)**:** dvaf008.

154. Kim H, Kim W-H, Kim Y-Y et al. Air Pollution and Central Nervous System Disease: A Review of the Impact of Fine Particulate Matter on Neurological Disorders. *Front Public Health* 2020; Volume 8 - 2020.

155. Armulik A, Genove G, Mae M et al. Pericytes regulate the blood-brain barrier. *Nature* 2010; 468(7323)**:** 557-561.

156. Zhang Y, Pei Y, Sun Y et al. AhR Agonistic Components in Urban Particulate Matter Regulate Astrocytic Activation and Function. *Environ Sci Technol* 2024; 58(10)**:** 4571-4580.

157. Ding ZB, Song LJ, Wang Q et al. Astrocytes: a double-edged sword in neurodegenerative diseases. *Neural Regen Res* 2021; 16(9)**:** 1702-1710.

158. Li B, Chang X, Liang X et al. The role of reactive astrocytes in neurotoxicity induced by ultrafine particulate matter. *Sci Total Environ* 2023; 867**:** 161416.

159. Ramirez-Mendoza AA, Mendoza-Magana ML, Ramirez-Herrera MA et al. Nitrooxidative Stress and Neuroinflammation Caused by Air Pollutants Are Associated with the Biological Markers of Neurodegenerative Diseases. *Antioxidants (Basel)* 2024; 13(3).

160. Zegeye MM, Lindkvist M, Fälker K et al. Activation of the JAK/STAT3 and PI3K/AKT pathways are crucial for IL-6 trans-signaling-mediated pro-inflammatory response in human vascular endothelial cells. *Cell Communication and Signaling* 2018; 16(1)**:** 55.

161. Wiggins-Dohlvik K, Merriman M, Shaji CA et al. Tumor necrosis factor-α disruption of brain endothelial cell barrier is mediated through matrix metalloproteinase-9. *The American Journal of Surgery* 2014; 208(6)**:** 954-960.

162. Aveleira CA, Lin C-M, Abcouwer SF et al. TNF-α Signals Through PKCζ/NF-κB to Alter the Tight Junction Complex and Increase Retinal Endothelial Cell Permeability. *Diabetes* 2010; 59(11)**:** 2872-2882.

163. Lee HG, Lee JH, Flausino LE et al. Neuroinflammation: An astrocyte perspective. *Sci Transl Med* 2023; 15(721)**:** eadi7828.

164. Blanco-Suarez E, Caldwell AL, Allen NJ. Role of astrocyte-synapse interactions in CNS disorders. *J Physiol* 2017; 595(6)**:** 1903-1916.

165. Gao C, Jiang J, Tan Y et al. Microglia in neurodegenerative diseases: mechanism and potential therapeutic targets. *Signal Transduct Target Ther* 2023; 8(1)**:** 359.

166. Hosli L, Zuend M, Bredell G et al. Direct vascular contact is a hallmark of cerebral astrocytes. *Cell Rep* 2022; 39(1)**:** 110599.

167. Diaz-Castro B, Robel S, Mishra A. Astrocyte Endfeet in Brain Function and Pathology: Open Questions. *Annu Rev Neurosci* 2023; 46**:** 101-121.

168. Schiera G, Di Liegro CM, Schiro G et al. Involvement of Astrocytes in the Formation, Maintenance, and Function of the Blood-Brain Barrier. *Cells* 2024; 13(2).

169. Michinaga S, Koyama Y. Dual Roles of Astrocyte-Derived Factors in Regulation of Blood-Brain Barrier Function after Brain Damage. *Int J Mol Sci* 2019; 20(3).

170. Argaw AT, Asp L, Zhang J et al. Astrocyte-derived VEGF-A drives blood-brain barrier disruption in CNS inflammatory disease. *J Clin Invest* 2012; 122(7)**:** 2454-2468.

171. Ronaldson PT, DeMarco KM, Sanchez-Covarrubias L et al. Transforming Growth Factor-β Signaling Alters Substrate Permeability and Tight Junction Protein Expression at the Blood-Brain Barrier during Inflammatory Pain. *J Cereb Blood Flow Metab* 2009; 29(6)**:** 1084-1098.

172. Kolliker-Frers R, Udovin L, Otero-Losada M et al. Neuroinflammation: An Integrating Overview of Reactive-Neuroimmune Cell Interactions in Health and Disease. *Mediators Inflamm* 2021; 2021**:** 9999146.

173. Chen Z, Liu P, Xia X et al. The underlying mechanism of PM2.5-induced ischemic stroke. *Environ Pollut* 2022; 310**:** 119827.

174. Onoda A, Kawasaki T, Tsukiyama K et al. Perivascular Accumulation of β-Sheet-Rich Proteins in Offspring Brain following Maternal Exposure to Carbon Black Nanoparticles. *Front Cell Neurosci* 2017; Volume 11 - 2017.

175. Onoda A, Takeda K, Umezawa M. Pretreatment with N-acetyl cysteine suppresses chronic reactive astrogliosis following maternal nanoparticle exposure during gestational period. *Nanotoxicology* 2017; 11(8)**:** 1012-1025.

176. Morrel J, Sukumaran K, Torgerson C et al. Outdoor Air Pollution, Perivascular Space Morphology, and Cognition in Preadolescence. *bioRxiv* 2025**:** 2025.2009.2026.678867.

177. Hussain R, Graham U, Elder A et al. Air pollution, glymphatic impairment, and Alzheimer's disease. *Trends Neurosci* 2025; (1878-108X (Electronic)).

178. Jankowska-Kieltyka M, Roman A, Nalepa I. The Air We Breathe: Air Pollution as a Prevalent Proinflammatory Stimulus Contributing to Neurodegeneration. *Front Cell Neurosci* 2021; Volume 15 - 2021.

179. Tuovinen T, Kananen J, Rajna Z et al. The variability of functional MRI brain signal increases in Alzheimer's disease at cardiorespiratory frequencies. *Sci Rep* 2020; 10(1)**:** 21559.

180. Wang BR, Shi JQ, Ge NN et al. PM2.5 exposure aggravates oligomeric amyloid beta-induced neuronal injury and promotes NLRP3 inflammasome activation in an in vitro model of Alzheimer's disease. *J Neuroinflammation* 2018; 15(1)**:** 132.

181. Kim RE, Shin CY, Han SH et al. Astaxanthin Suppresses PM2.5-Induced Neuroinflammation by Regulating Akt Phosphorylation in BV-2 Microglial Cells. *Int J Mol Sci* 2020; 21(19).

182. Bos I, De Boever P, Emmerechts J et al. Changed gene expression in brains of mice exposed to traffic in a highway tunnel. *Inhal Toxicol* 2012; 24(10)**:** 676-686.

183. Charlton T, Prowse N, McFee A et al. Brain-derived neurotrophic factor (BDNF) has direct anti-inflammatory effects on microglia. *Front Cell Neurosci* 2023; 17**:** 1188672.

184. Chen X, Guo J, Huang Y et al. Urban airborne PM(2.5)-activated microglia mediate neurotoxicity through glutaminase-containing extracellular vesicles in olfactory bulb. *Environ Pollut* 2020; 264**:** 114716.

185. Haruwaka K, Ikegami A, Tachibana Y et al. Dual microglia effects on blood brain barrier permeability induced by systemic inflammation. *Nat Commun* 2019; 10(1)**:** 5816.

186. Coburn JL, Cole TB, Dao KT et al. Acute exposure to diesel exhaust impairs adult neurogenesis in mice: prominence in males and protective effect of pioglitazone. *Arch Toxicol* 2018; 92(5)**:** 1815-1829.

187. Barros MH, Hauck F, Dreyer JH et al. Macrophage polarisation: an immunohistochemical approach for identifying M1 and M2 macrophages. *PLoS One* 2013; 8(11)**:** e80908.

188. Ronaldson PT, Davis TP. Regulation of blood-brain barrier integrity by microglia in health and disease: A therapeutic opportunity. *J Cereb Blood Flow Metab* 2020; 40(1_suppl)**:** S6-S24.

189. Araujo JE, Jorge S, Santos HM et al. Proteomic changes driven by urban pollution suggest particulate matter as a deregulator of energy metabolism, mitochondrial activity, and oxidative pathways in the rat brain. *Sci Total Environ* 2019; 687**:** 839-848.

190. Sama P, Long TC, Hester S et al. The cellular and genomic response of an immortalized microglia cell line (BV2) to concentrated ambient particulate matter. *Inhal Toxicol* 2007; 19(13)**:** 1079-1087.

191. Gimeno-Bayon J, Lopez-Lopez A, Rodriguez MJ et al. Glucose pathways adaptation supports acquisition of activated microglia phenotype. *J Neurosci Res* 2014; 92(6)**:** 723-731.

192. Ribeiro DE, Roncalho AL, Glaser T et al. P2X7 Receptor Signaling in Stress and Depression. *Int J Mol Sci* 2019; 20(11).

193. Liu C, She Y, Huang J et al. HMGB1-NLRP3-P2X7R pathway participates in PM(2.5)-induced hippocampal neuron impairment by regulating microglia activation. *Ecotoxicol Environ Saf* 2022; 239**:** 113664.

194. Bodin P, Burnstock G. Purinergic signalling: ATP release. *Neurochem Res* 2001; 26(8-9)**:** 959-969.

195. Zeng X, Liu D, Wu W et al. PM(2.5) exposure inducing ATP alteration links with NLRP3 inflammasome activation. *Environ Sci Pollut Res Int* 2022; 29(17)**:** 24445-24456.

196. Ye M, Yang J, Li J et al. Progress in Mechanisms, Pathways and Cohort Studies About the Effects of PM2.5 Exposure on the Central Nervous System. *Rev Environ Contam Toxicol* 2023; 261(7).

197. Liu LR, Liu JC, Bao JS et al. Interaction of Microglia and Astrocytes in the Neurovascular Unit. *Front Immunol* 2020; 11**:** 1024.

198. Garland EF, Hartnell IJ, Boche D. Microglia and Astrocyte Function and Communication: What Do We Know in Humans? *Front Neurosci* 2022; 16**:** 824888.

199. Wu Y, Eisel ULM. Microglia-Astrocyte Communication in Alzheimer's Disease. *J Alzheimers Dis* 2023; 95(3)**:** 785-803.

200. Zhang Y, Lian L, Fu R et al. Microglia: The Hub of Intercellular Communication in Ischemic Stroke. *Front Cell Neurosci* 2022; 16**:** 889442.

201. Keren-Shaul H, Spinrad A, Weiner A et al. A Unique Microglia Type Associated with Restricting Development of Alzheimer's Disease. *Cell* 2017; 169(7)**:** 1276-1290 e1217.

202. Fairless R, Bading H, Diem R. Pathophysiological Ionotropic Glutamate Signalling in Neuroinflammatory Disease as a Therapeutic Target. *Front Neurosci* 2021; 15**:** 741280.

203. Lai TW, Zhang S, Wang YT. Excitotoxicity and stroke: identifying novel targets for neuroprotection. *Prog Neurobiol* 2014; 115**:** 157-188.

204. Szepesi Z, Manouchehrian O, Bachiller S et al. Bidirectional Microglia-Neuron Communication in Health and Disease. *Front Cell Neurosci* 2018; 12**:** 323.

205. Bernardo-Castro S, Sousa JA, Bras A et al. Pathophysiology of Blood-Brain Barrier Permeability Throughout the Different Stages of Ischemic Stroke and Its Implication on Hemorrhagic Transformation and Recovery. *Front Neurol* 2020; 11**:** 594672.

206. Prinz M, Priller J. The role of peripheral immune cells in the CNS in steady state and disease. *Nat Neurosci* 2017; 20(2)**:** 136-144.

207. Barar J, Rafi MA, Pourseif MM et al. Blood-brain barrier transport machineries and targeted therapy of brain diseases. *Bioimpacts* 2016; 6(4)**:** 225-248.

208. Saib S, Delavenne X. Inflammation Induces Changes in the Functional Expression of P-gp, BCRP, and MRP2: An Overview of Different Models and Consequences for Drug Disposition. In: *Pharmaceutics*, 2021. p 1544.

209. Wittmann G, Mohácsik P, Balkhi MY et al. Endotoxin-induced inflammation down-regulates l-type amino acid transporter 1 (LAT1) expression at the blood–brain barrier of male rats and mice. *Fluids Barriers CNS* 2015; 12(1)**:** 21.

210. Villarroel F, Ramírez E, Ponce N et al. Impact of PM2.5 Emitted by Wood Smoke on the Expression of Glucose Transporter 1 (GLUT1) and Sodium-Dependent Vitamin C Transporter 2 (SVCT2) in the Rat Placenta: A Pregestational and Gestational Exposure Study. In: *Antioxidants*, 2025. p 1050.

211. Abbas I, Saint-Georges F, Billet S et al. Air pollution particulate matter (PM2.5)-induced gene expression of volatile organic compound and/or polycyclic aromatic hydrocarbon-metabolizing enzymes in an in vitro coculture lung model. *Toxicol In Vitro* 2009; 23(1)**:** 37-46.

212. Ali H, Hanna MM, Alziny N et al. The impact of the exposome on cytochrome P450-mediated drug metabolism. *Front Pharmacol* 2025; Volume 16 - 2025.

213. Refsnes M, Skuland T, Jørgensen R et al. Role of different mechanisms in pro-inflammatory responses triggered by traffic-derived particulate matter in human bronchiolar epithelial cells. *Part Fibre Toxicol* 2023; 20(1)**:** 31.

214. Connick JP, Stepter AA, Cawley GF et al. Environmentally persistent free radicals lead to selective inhibition of CYP1 monooxygenase activities, and increased production of reactive oxygen species by reaction uncoupling. *Front Public Health* 2025; Volume 13 - 2025.

215. Green R, Broadwin R Fau - Malig B, Malig B Fau - Basu R et al. Long- and Short-term Exposure to Air Pollution and Inflammatory/Hemostatic Markers in Midlife Women. 2016; (1531-5487 (Electronic)).

216. Lanki T, Hampel R, Tiittanen P et al. Air Pollution from Road Traffic and Systemic Inflammation in Adults: A Cross-Sectional Analysis in the European ESCAPE Project. *Environ Health Perspect* 2015; 123(8)**:** 785-791.

217. Michikawa T, Okamura T, Nitta H et al. Cross-sectional association between exposure to particulate matter and inflammatory markers in the Japanese general population: NIPPON DATA2010. *Environ Pollut* 2016; 213**:** 460-467.

218. Niu J, Liberda EN, Qu S et al. The Role of Metal Components in the Cardiovascular Effects of PM2.5. *PLOS ONE* 2013; 8(12)**:** e83782.

219. Viehmann A, Hertel S, Fuks K et al. Long-term residential exposure to urban air pollution, and repeated measures of systemic blood markers of inflammation and coagulation. *Occup Environ Med* 2015; 72(9)**:** 656.

220. Pope CA, Bhatnagar A, McCracken JP et al. Exposure to Fine Particulate Air Pollution Is Associated With Endothelial Injury and Systemic Inflammation. *Circulation Research* 2016; 119(11)**:** 1204-1214.

221. Tsai D-H, Amyai N, Marques-Vidal P et al. Effects of particulate matter on inflammatory markers in the general adult population. *Part Fibre Toxicol* 2012; 9(1)**:** 24.

222. Wolf K, Popp A, Schneider A et al. Association Between Long-term Exposure to Air Pollution and Biomarkers Related to Insulin Resistance, Subclinical Inflammation, and Adipokines. *Diabetes* 2016; 65(11)**:** 3314-3326.

223. Siponen T, Yli-Tuomi T, Aurela M et al. Source-specific fine particulate air pollution and systemic inflammation in ischaemic heart disease patients. *Occup Environ Med* 2015; 72(4)**:** 277.

224. Cai Y, Zhang B, Ke W et al. Associations of Short-Term and Long-Term Exposure to Ambient Air Pollutants With Hypertension. *Hypertension* 2016; 68(1)**:** 62-70.

225. Yang B-Y, Qian Z, Howard SW et al. Global association between ambient air pollution and blood pressure: A systematic review and meta-analysis. *Environ Pollut* 2018; 235**:** 576-588.

226. Farhadi Z, Abulghasem Gorgi H, Shabaninejad H et al. Association between PM2.5 and risk of hospitalization for myocardial infarction: a systematic review and a meta-analysis. *BMC Public Health* 2020; 20(1)**:** 314.

227. Madrigano J, Kloog I, Goldberg R et al. Long-term Exposure to PM2.5 and Incidence of Acute Myocardial Infarction. *Environ Health Perspect* 2013; 121(2)**:** 192-196.

228. Rhinehart ZJ, Kinnee E, Essien UR et al. Association of Fine Particulate Matter and Risk of Stroke in Patients With Atrial Fibrillation. *JAMA Netw Open* 2020; 3(9)**:** e2011760-e2011760.

229. Yuan S, Wang J, Jiang Q et al. Long-term exposure to PM2.5 and stroke: A systematic review and meta-analysis of cohort studies. *Environ Res* 2019; 177**:** 108587.

230. O'Donnell MJ, Fang J, Mittleman MA et al. Fine Particulate Air Pollution (PM2.5) and the Risk of Acute Ischemic Stroke. *Epidemiology* 2011; 22(3).

231. Wang M, Zhou T, Song Y et al. Joint exposure to various ambient air pollutants and incident heart failure: a prospective analysis in UK Biobank. *Eur Heart J* 2021; 42(16)**:** 1582-1591.

232. Liu H, Tian Y, Song J et al. Effect of Ambient Air Pollution on Hospitalization for Heart Failure in 26 of China's Largest Cities. *The American Journal of Cardiology* 2018; 121(5)**:** 628-633.

233. Wang F, Ahat X, Liang Q et al. The relationship between exposure to PM2.5 and atrial fibrillation in older adults: A systematic review and meta-analysis. *Sci Total Environ* 2021; 784**:** 147106.

234. Newby DE, Mannucci PM, Tell GS et al. Expert position paper on air pollution and cardiovascular disease. *Eur Heart J* 2015; 36(2)**:** 83-93.

235. Vlaanderen J, Vermeulen R, Whitaker M et al. Impact of long-term exposure to PM2.5 on peripheral blood gene expression pathways involved in cell signaling and immune response. *Environ Int* 2022; 168**:** 107491.

236. Marín-Palma D, Fernandez GJ, Ruiz-Saenz J et al. Particulate matter impairs immune system function by up-regulating inflammatory pathways and decreasing pathogen response gene expression. *Sci Rep* 2023; 13(1)**:** 12773.

237. Kim B, Blam K, Elser H et al. Ambient Air Pollution and the Severity of Alzheimer Disease Neuropathology. *JAMA Neurol* 2025; 82(11)**:** 1153-1161.

238. Hajat A, Park C, Adam C et al. Air pollution and plasma amyloid beta in a cohort of older adults: Evidence from the Ginkgo Evaluation of Memory study. *Environ Int* 2023; 172**:** 107800.

239. Alemany S, Crous-Bou M, Vilor-Tejedor N et al. Associations between air pollution and biomarkers of Alzheimer’s disease in cognitively unimpaired individuals. *Environ Int* 2021; 157**:** 106864.

240. Ma Y-H, Chen H-S, Liu C et al. Association of Long-term Exposure to Ambient Air Pollution With Cognitive Decline and Alzheimer’s Disease–Related Amyloidosis. *Biological Psychiatry* 2023; 93(9)**:** 780-789.

241. Calderón-Garcidueñas L, Mukherjee PS, Waniek K et al. Non-Phosphorylated Tau in Cerebrospinal Fluid is a Marker of Alzheimer’s Disease Continuum in Young Urbanites Exposed to Air Pollution. *J Alzheimers Dis* 2018; 66(4)**:** 1437-1451.

242. Iaccarino L, La Joie R, Lesman-Segev OH et al. Association Between Ambient Air Pollution and Amyloid Positron Emission Tomography Positivity in Older Adults With Cognitive Impairment. *JAMA Neurol* 2021; 78(2)**:** 197-207.

243. Casey E, Li Z, Liang D et al. Association between Fine Particulate Matter Exposure and Cerebrospinal Fluid Biomarkers of Alzheimer’s Disease among a Cognitively Healthy Population-Based Cohort. *Environ Health Perspect* 2024; 132(4)**:** 047001.

244. Fu P, Guo X, Cheung FMH et al. The association between PM2.5 exposure and neurological disorders: A systematic review and meta-analysis. *Sci Total Environ* 2019; 655**:** 1240-1248.

245. Wang Y, Wu J, Hu R et al. Airborne PM2.5 and Parkinson's disease: An updated meta-analysis. *J Environ Occup Med* 2024; 41(2)**:** 168-174.

246. Shin S, Burnett RT, Kwong JC et al. Effects of ambient air pollution on incident Parkinson’s disease in Ontario, 2001 to 2013: a population-based cohort study. *Int J Epidemiol* 2018; 47(6)**:** 2038-2048.

247. Jahanshahi B, McVicar D, Rowland N. Exposure to ambient air pollution and onset of Parkinson’s disease in a large cohort study. *NPJ Parkinsons Dis* 2025; 11(1)**:** 291.

248. Wei Y, Wang Y, Di Q et al. Short term exposure to fine particulate matter and hospital admission risks and costs in the Medicare population: time stratified, case crossover study. *BMJ* 2019; 367**:** l6258.

249. Krzyzanowski B, Mullan AF, Turcano P et al. Air Pollution and Parkinson Disease in a Population-Based Study. *JAMA Netw Open* 2024; 7(9)**:** e2433602-e2433602.

250. Yuan X, Yang Y, Liu C et al. Fine Particulate Matter Triggers α-Synuclein Fibrillization and Parkinson-like Neurodegeneration. *Mov Disord* 2022; 37(9)**:** 1817-1830.

251. Wang Y, Li C, Zhang X et al. Exposure to PM2.5 aggravates Parkinson’s disease via inhibition of autophagy and mitophagy pathway. *Toxicol* 2021; 456**:** 152770.

252. George S, Rey NL, Tyson T et al. Microglia affect α-synuclein cell-to-cell transfer in a mouse model of Parkinson’s disease. *Mol Neurodegener* 2019; 14(1)**:** 34.

253. Mercado G, Kaeufer C, Richter F et al. Infections in the Etiology of Parkinson’s Disease and Synucleinopathies: A Renewed Perspective, Mechanistic Insights, and Therapeutic Implications. *J Parkinsons Dis* 2024; 14(7)**:** 1301-1329.

254. Yuan S, Huang X, Zhang L et al. Associations of air pollution with all-cause dementia, Alzheimer’s disease, and vascular dementia: a prospective cohort study based on 437,932 participants from the UK biobank. *Front Neurosci* 2023; Volume 17 - 2023.

255. Wilker EH, Osman M, Weisskopf MG. Ambient air pollution and clinical dementia: systematic review and meta-analysis. *BMJ* 2023; 381**:** e071620.

256. Gong Y, Zhang X, Zhao X et al. Global ambient particulate matter pollution and neurodegenerative disorders: a systematic review of literature and meta-analysis. *Environ Sci Pollut Res* 2023; 30(14)**:** 39418-39430.

257. Saeidnia S, Manayi A, Abdollahi M. From in vitro Experiments to in vivo and Clinical Studies; Pros and Cons. *Current Drug Discovery Technologies* 2015; 12(4)**:** 218-224.

258. Hajal C, Offeddu GS, Shin Y et al. Engineered human blood–brain barrier microfluidic model for vascular permeability analyses. *Nat Protoc* 2022; 17(1)**:** 95-128.

259. Hameed S, Zhao J, Zare RN. Ambient PM particles reach mouse brain, generate ultrastructural hallmarks of neuroinflammation, and stimulate amyloid deposition, tangles, and plaque formation. *Talanta Open* 2020; 2**:** 100013.

260. Heidari Nejad S, Takechi R, Mullins BJ et al. The effect of diesel exhaust exposure on blood–brain barrier integrity and function in a murine model. *J Appl Toxicol* 2015; 35(1)**:** 41-47.

261. Calderón-Garcidueñas L, Solt AC, Henríquez-Roldán C et al. Long-term Air Pollution Exposure Is Associated with Neuroinflammation, an Altered Innate Immune Response, Disruption of the Blood-Brain Barrier, Ultrafine Particulate Deposition, and Accumulation of Amyloid β-42 and α-Synuclein in Children and Young Adults. *Toxicol Pathol* 2008; 36(2)**:** 289-310.

262. Vanbrabant K, Van Dam D, Bongaerts E et al. Accumulation of Ambient Black Carbon Particles Within Key Memory-Related Brain Regions. *JAMA Netw Open* 2024; 7(4)**:** e245678-e245678.

263. Nephew BC, Nemeth A, Hudda N et al. Traffic-related particulate matter affects behavior, inflammation, and neural integrity in a developmental rodent model. *Environ Res* 2020; 183**:** 109242.

264. Pan D, Xu Y, Wang X et al. Evaluation the in vivo behaviors of PM2.5 in rats using noninvasive PET imaging with mimic particles. *Chemosphere* 2023; 339**:** 139663.

265. Li D, Li Y, Li G et al. Fluorescent reconstitution on deposition of PM2.5 in lung and extrapulmonary organs. *Proc Natl Acad Sci U S A* 2019; 116(7)**:** 2488-2493.
